# Analyzing the Impact of Rehabilitation Utilizing Neurofunctional Exercises on the Functional Status of Stroke Patients

**DOI:** 10.3390/jcm13206271

**Published:** 2024-10-21

**Authors:** Rafał Studnicki, Karolina Studzińska, Tomasz Adamczewski, Rita Hansdorfer-Korzon, Maciek Krawczyk

**Affiliations:** 1 Department of Physiotherapy, Medical University of Gdańsk, 7 Dębinki Street, 80-211 Gdańsk, Poland; karolina.studzinska@gumed.edu.pl (K.S.); rita.hansdorfer-korzon@gumed.edu.pl (R.H.-K.); 2 Central University Hospital, Outpatient Clinic, Devision Physiotherapy, Medical University of Łódź, St. Pomorska 251, 92-213 Łódź, Poland; tadamcz@csk.umed.pl; 3 IInd Deparment of Neurology, Institute of Psychiatry and Neurology, 02-957 Warsaw, Poland; krawiec@awf.edu.pl; 4 Faculty of Rehabilitation, University of Physical Education, 00-968 Warsaw, Poland

**Keywords:** stroke, rehabilitation, quality of life, healthcare

## Abstract

**Background/Objectives**: Physical rehabilitation based on neurofunctional exercises can have a positive impact on restoring functionality and enhancing the quality of life of these individuals. Therefore, the purpose of this study is to analyze the effects of rehabilitation, including neurofunctional exercises, on the functional status of stroke patients. **Methods**: The cohort study design included 102 male and female participants: 51 patients underwent physiotherapy rehabilitation including neurofunctional exercises (SG), while the other 51 did not follow a rehabilitation program based on neurofunctional exercises (CG). The participants were assessed twice: once during their stay in the early neurology department after the first stroke, and again six months later. The assessments were conducted using the Barthel Scale (BS), the Rankin Scale (RS), and the National Institutes of Health Stroke Scale (NIHSS). **Results**: Baseline comparisons revealed significantly greater BS (*p* = 0.001) in the CG compared to the SG. Conversely, the SG had a significantly higher NIHSS than the CG at baseline (*p* = 0.001), as well as higher RS (*p* < 0.001). Within the SG, there were significant increases in BS (*p* < 0.001), while no significant differences were found between baseline and post 6 months in RS (*p* = 0.537) and NIHSS (*p* = 0.475). Regarding the CG, significant increases were observed in BS (*p* = 0.005) and NIHSS (*p* < 0.001), while no significant differences were found in RS (*p* = 0.335). **Conclusions**: In conclusion, this study reveals that incorporating neurofunctional exercises does not appear to play a significant role in the patients’ progress. The controlled group, engaged in home-based activities, showed greater improvements in their condition.

## 1. Introduction

Stroke, a frequently diagnosed neurological disease, is characterized by focal damage to the central nervous system [1]. Stroke can cause a wide range of functional limitations depending on the area of the brain affected and the severity of the event. Common limitations include motor deficits such as hemiplegia or hemiparesis, where one side of the body experiences weakness or complete paralysis, impairing mobility and coordination. Cognitive impairments can also occur, affecting memory, attention, and executive functions. Additionally, stroke can lead to speech and language difficulties, including aphasia, which hampers the ability to communicate effectively. Sensory disturbances, such as numbness or altered sensation, and visual deficits like partial blindness or visual field loss, are also frequent. These limitations can significantly impact a person’s ability to perform daily activities and maintain independence [2]. The global stroke burden has risen significantly between 1990 and 2021, alongside an increase in the impact of various risk factors [3]. To effectively reduce this burden, it is critical to implement urgent, widespread measures for better stroke monitoring, prevention—particularly focusing on blood pressure control, lifestyle changes, and environmental influences—along with improvements in acute care and rehabilitation [3].

Physical activity plays a crucial role in improving outcomes for patients recovering from stroke. Regular physical exercise enhances neuroplasticity, aiding in the recovery of motor and cognitive functions. It also helps to improve cardiovascular fitness, which can reduce the risk of recurrent strokes. Engaging in physical activities, such as strength training, aerobic exercises, and balance exercises, can improve muscle strength, flexibility, and coordination, thereby enhancing mobility and reducing the risk of falls [4]. Moreover, physical activity is linked to improvements in mood and reduction in post-stroke depression, contributing to overall psychological well-being and quality of life. Structured rehabilitation programs that include tailored physical activity regimens are essential in optimizing recovery and facilitating a return to daily activities. Finally, engaging in physical activity lowers the risk of both an initial stroke and a recurrent one.

Currently, hospital rehabilitation is short-term, shifting the focus from hospital-based care to the local environment. This change has increased the demand for home physiotherapy solutions and encourages patients to engage in activities that improve their physical fitness and quality of life [5]. A study conducted by French et al. shows that repetitive performance of certain daily activities improves performance and activities of daily living (ADL) in stroke survivors [6]. According to research by Aguiar et al., moderate physical activity and exercise, averaging 40 min per day, including everyday activities, are recommended [7]. These activities can clearly improve the prognosis of patients. For instance, a systematic review revealed that increased physical activity after a stroke enhances cognitive performance [8]. Moreover, another review confirmed that exercise interventions beneficially address four of the top ten shared research priorities: cognition, arm function, balance and gait, and exercise programs. Additionally, emerging evidence supports the potential benefits of exercise in addressing two more priorities: fatigue and confidence [9].

However, stroke patients often find the difficulty and frequency of activities challenging, yet they still express a desire to participate in social life [7]. Nearly 30% of patients have functional limitations in basic activities, and 50% require support to perform basic ADLs [10,11]. This is supported by a study by Hildebrand et al., which suggests that individuals who have experienced a mild stroke do not continue the demanding recreational activities they engaged in before the stroke [12]. This trend may apply to over 50% of stroke patients who reported being active during their free time [13]. Studies have shown that stroke patients living in local communities exhibit reduced physical activity levels [14]. Research has revealed that participation in physical activity is influenced by a variety of factors, including the individual’s clinical condition, as well as personal and social factors, such as cultural differences [15]. For example, older stroke survivors may be at a higher risk of physical inactivity [16]. However, by targeting potentially modifiable factors such as physical function, depression, self-efficacy, and quality of life, it is possible to increase physical activity levels among stroke survivors [16]. For example, research suggests that while rehabilitation services primarily target patients, recognizing patients as integral members of extended families, communities, and broader society can foster greater long-term adherence to a physically active lifestyle. Since social factors significantly impact motivation for physical activity, it is imperative to involve all these stakeholders to facilitate the transition to a normal life after a stroke [17].

Despite advancements in stroke rehabilitation, there remains a significant gap in understanding the comparative effectiveness of rehabilitation interventions that utilize neurofunctional exercises versus those that utilize non-neurofunctional exercises on the functionality levels of stroke patients. While existing research emphasizes the benefits of physical activity and tailored rehabilitation programs in enhancing motor and cognitive functions, reducing depression, and improving quality of life, there is limited exploration into the outcomes of stroke patients not exposed to formal rehabilitation, namely those exposed to neurofunctional exercises [18]. Our study seeks to address this gap by investigating the impact of rehabilitation that utilizes neurofunctional exercises versus standard rehabilitation that does not utilize neurofunctional exercises on functionality levels in stroke survivors. By elucidating the potential differences in outcomes between these two groups, our research aims to provide valuable insights into the effectiveness of rehabilitation interventions and contribute to the development of more targeted and evidence-based rehabilitation strategies. This study is particularly pertinent given the increasing demand for rehabilitation solutions and the challenges faced by stroke patients in maintaining physical activity levels post-discharge.

## 2. Materials and Methods

### 2.1. Study Design

This study followed a prospective cohort design. Adhering to the standards for reporting such studies, we followed the STROBE guidelines for cohort designs [19]. A pre–post repeated measures approach was employed, where patients suffering from stroke were assessed using the Barthel Scale (BS), Rankin Scale (RS), and National Institutes of Health Stroke Scale (NIHSS) during their hospitalization after the stroke. The same assessments were repeated six months later. Participants were categorized into two groups: those who were enrolled in rehabilitation between the stroke event and the second assessment period, and those who were not.

### 2.2. Ethical Standards

The Independent Bioethics Committee for Scientific Research of the Medical University of Gdańsk approved the present study (Resolution No. NKEBN/76-271/2022), which was conducted in cooperation with the Department of Neurological Rehabilitation in Gdańsk. Participants were informed about the study’s objectives and procedures and provided their informed consent for inclusion. This study was conducted in accordance with the ethical principles of the Declaration of Helsinki.

### 2.3. Participants

An a priori sample size estimation was conducted for a repeated measures ANOVA with within-subjects and between-subjects factors. The parameters included a power of 0.85, an effect size of 0.2, and a significance level of *p* = 0.05, for 2 groups and 2 measurements. Using G*Power (version 3.1.9.6., Universität Düsseldorf, Düsseldorf, Germany), the recommended sample size was determined to be 60 participants.

Patients diagnosed with cerebral stroke and hospitalized in the neurology department of Saint Wojciech Specialist Hospital in Gdańsk, with a total of 1007 patients, were considered for the preliminary analysis. Initial qualification for the study was conducted via telephone interviews, during which a survey was administered to exclude patients unlikely to suffer from spasticity. The survey included questions on the following: difficulty in raising the arm above the head, the ability to grasp an object independently, and difficulty in walking.

The inclusion criteria for the study were as follows: (i) first ischemic or hemorrhagic stroke in life, diagnosed through physical examination; (ii) paresis of one side of the body.

Spasticity of at least 2 points according to the Modified Ashworth Scale (MAS) in at least one muscle group; (iii) muscle weakness and abnormal reflexes; (iv) informed consent to participate in the study after being provided with information about the study; (v) age over 18 years; (vi) no prior medical rehabilitation in a rehabilitation department. Exclusion criteria included: (i) diagnosis of transient ischemic attack (TIA); (ii) a confirmed second or subsequent stroke; (iii) presence of additional diseases that could affect the development of spasticity, such as cancer, heart attack, or apallic state; (iv) lack of consent from the patient or family; (v) medical rehabilitation conducted in a rehabilitation department.

**Figure 1:**
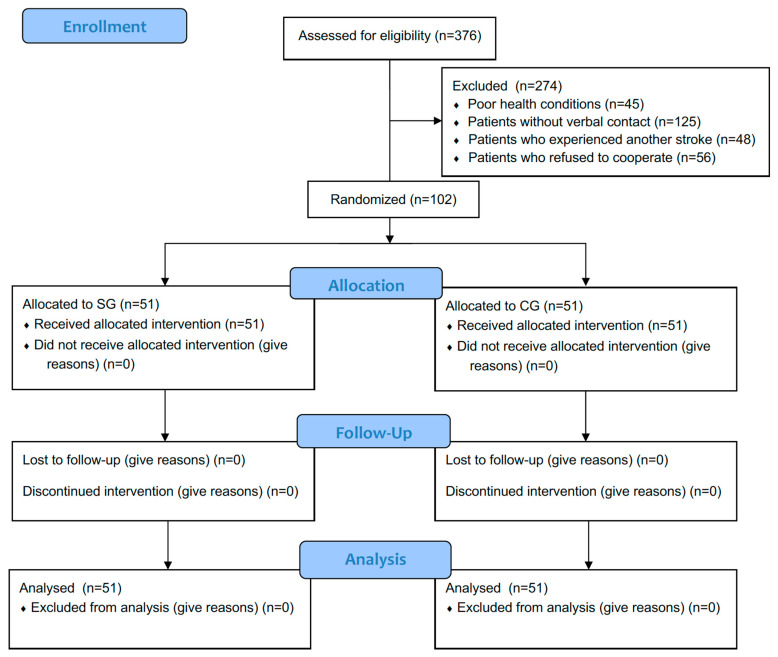
Flowchart of participants.

Diagnostic visits were carried out by a physiotherapist at the patient’s place of residence. Patients were excluded if they did not have spasticity (*n* = 456) or if contact was lost for various reasons (*n* = 175). Initially, 376 patients were qualified, but further exclusions were made: patients with poor health conditions (*n* = 45), patients without verbal contact (*n* = 125), patients who experienced another stroke (*n* = 48), and patients who refused to cooperate (*n* = 56). Ultimately, 102 patients (Figure 1) with spasticity after stroke were included in the study after obtaining informed consent from the patient or legal guardian.

Of the 102 participants, 51 (20 men and 31 women; average age 72.2 ± 9.9 years) were assigned to the group participating in rehabilitation using neurofunctional exercises (SG), while the other 51 (26 men and 25 women; average age 69.5 ± 10.8 years) performed standard exercises without the neurophysiological method (CG).

### 2.4. Independent Variables

The patients were categorized into two groups according to the type of treatment: Study Group (SG): Comprising 51 patients who underwent hospital rehabilitation within six months of their initial stroke. Following discharge from the early stroke neurology unit, these patients awaited placement in a rehabilitation hospital. Subsequently, they underwent inpatient rehabilitation in the rehabilitation department, which encompassed standard rehabilitation protocols including neurofunctional exercises with elements of Proprioceptive Neuromuscular Facilitation (PNF) and Bobath Neurodevelopmental Therapy (BNT) for upper and lower limb rehabilitation, gait training, self-care training, and ergometer training. In PNF therapy, combined patterns were used, such as flexion–adduction–external rotation and extension–abduction–internal rotation of the paretic upper and lower limbs. PNF patterns were performed from a lying position to a half-standing position, constantly monitoring the state of the trunk position and the movement of the pattern. The proposed NDT–Bobath therapy method was in line with the functional level and deficit of the patients. The exercises were aimed at stimulating the body’s balance response through repetition, challenging and strengthening it. The positions used, such as lying or sitting, were used to inhibit tension and abnormal movement patterns, facilitate normal movement and stimulate muscle inactivity. The SG underwent daily standard exercises involving passive exercises for the upper and lower limbs, gait training, self-care training, and ergometer training for a duration of 6 weeks. The sessions lasted 45 min per day. The duration of stay in the rehabilitation ward was six weeks.

Control Group (CG): Consisted of 51 patients who did not undergo any hospital rehabilitation within six months of their first stroke. After leaving the early stroke neurology unit, these patients remained at home. They were encouraged to stay active by their family members (children, spouse) and guardian. CG were treated daily with standard exercises (passive exercises of upper and lower limbs, gait training and ergometer training, 6 weeks of therapy) without neurophysiological method (PNF, NDT–Bobath).

### 2.5. Procedures

The evaluators (neurologists) conducted the surveys after the stroke (during their stay in the early neurology department) and six months post-stroke using the three previously mentioned scales (BS, RS, NIHSS). Each survey was administered individually by the same group of evaluators, who were already experts in applying these scales. The surveys were conducted in a quiet room with no other participants present.

### 2.6. Barthel Scale

The BS is an international scale for assessing a patient’s fitness and care needs [20]. The scale has demonstrated excellent inter-rater reliability for standard administration following stroke and has been validated as an appropriate outcome measure for stroke trials and clinical practice, as evidenced by a systematic review and meta-analysis [21]. The index evaluates the patient’s level of dexterity and independence in ten basic areas: eating, moving and sitting, maintaining personal hygiene, using the toilet, washing and bathing the entire body, moving on flat surfaces, climbing and descending stairs, dressing and undressing, controlling stool and the anal sphincter, and controlling urine and the bladder sphincter. The scores for each item range from 0 to 3, although in some cases they may range from 0 to 1 or from 0 to 2 [20]. The interpretation of scores is as follows: 0–20 points—complete dependence; 21–80 points—assistance from others needed; 81–100 points—the patient can function independently with support from others [20]. The score for each patient was obtained at both assessment moments and was utilized for further data processing.

### 2.7. Rankin Scale

The RS is a clinician-reported measure of global disability that has been widely applied for evaluating recovery from stroke [22]. The RS provides a standardized and reliable method [23] for clinicians to communicate and document the functional status of stroke survivors, facilitating consistent evaluation and comparison across different healthcare settings and research studies. The scale categorizes a patient’s level of disability into seven grades, ranging from 0 (no symptoms) to 5 (severe disability: bedridden, incontinent, and requiring constant nursing care and attention). The scale evaluates the patient’s ability to carry out activities of daily living independently, such as self-care, mobility, and communication. Scores were obtained for each patient at both assessment moments and were used for further data analysis.

### 2.8. National Institutes of Health Stroke Scale

The NIHSS is a systematic, standardized, and reliable assessment tool used to evaluate the severity of stroke-related neurological deficits [24,25]. The NIHSS is used both in acute settings to assess initial stroke severity and over time to monitor changes in a patient’s condition, making it a critical tool in stroke management and research. It comprises 15 items that measure various aspects of brain function, including consciousness level, gaze, visual fields, facial palsy, motor strength, limb ataxia, sensory loss, language, speech, and attention. Each item is scored on a scale, with higher scores indicating greater impairment. The total score ranges from 0 to 42, with scores stratified to predict stroke outcomes and guide clinical decision-making. A lower score suggests a less severe stroke and a better prognosis, while a higher score indicates more significant neurological damage and a potentially worse outcome. Scores range from 0 (normal) to 42 points (where: 1–4—minor stroke, 5–15—moderate stroke, 16–20—moderate to severe stroke, 21–42—severe stroke).

### 2.9. Statistical Procedures

Descriptive statistics were generated, including means and standard deviations. Prior to conducting inferential statistics, the sample’s normality was assessed and not confirmed with the Kolmogorov–Smirnov test (*p* < 0.05). Similarly, the assumption of homogeneity was not observed using Levene’s test (*p* < 0.05). Considering the absence of the assumption of movement in the parametric statistics, the non-parametric statistics were implemented. The Mann–Whitney U test was utilized to compare the groups at baseline and after 6 months post-stroke. Additionally, the Wilcoxon signed-rank test was employed to analyze the repeated measures within each group. The effect size was calculated using the *r* value, which is derived by dividing the z-value of the Mann–Whitney U test by the square root of N [26]. Statistical analyses were conducted using JASP software (version 0.18.3, University of Amsterdam, Amsterdam, The Netherlands), with a significance threshold set at *p* < 0.05.

## 3. Results

Table 1 presents the descriptive statistics of the demographic information and the rehabilitation scales between groups. Baseline comparisons revealed significantly greater BS (U = 820.5; Z = −3.233; *p* = 0.001) in the CG compared to the SG. Conversely, the SG had a significantly higher NIHSS than the CG at baseline (U = 647.5; Z = −4.406; *p* = 0.001), as well as higher RS (U = 811.5; Z = −3.342; *p* < 0.001).

**Figure 2:**
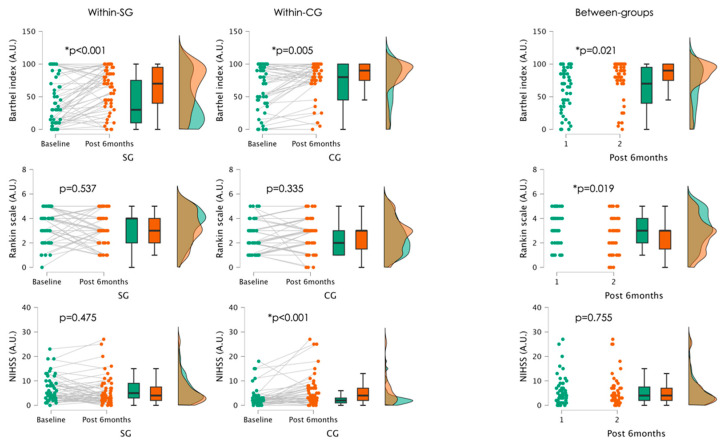
Raincloud plots describing the within and between-group differences between moments of assessment. NIHSS: National Institutes of Health Stroke Scale; A.U.: arbitrary units. SG: experimental group; CG: control group.

Figure 2 presents the descriptive statistics and the comparisons both within and between groups.

Post-intervention analysis revealed that the BS was significantly higher in the CG (SG: 63.5 ± 32.9 vs. CG: 78.1 ± 28.0; Z = −2.308; *p* = 0.021), while the RS was significantly higher in the SG compared to the CG (SG: 3.3 ± 1.3 vs. CG: 2.6 ± 1.4; Z = −2.348; *p* = 0.019). No significant differences were found in NIHSS scores between the two groups (SG: 5.9 ± 6.0 vs. CG: 5.8 ± 6.3; Z = −0.313; *p* = 0.755).

Within the SG, there were significant increases in BS (Z = −3.861; *p* < 0.001), while no significant differences were found between baseline and post 6 months in RS (Z = −0.617; *p* = 0.537) and NIHSS (Z = −0.715; *p* = 0.475). Regarding the CG, significant increases were observed in BS (Z = −2.821; *p* = 0.005) and NIHSS (Z = −3.954; *p* < 0.001), while no significant differences were found in RS (Z = −0.964; *p* = 0.335).

## 4. Discussion

The current prospective cohort study revealed that stroke patients who participated in neurofunctional exercises did not experience significant benefits in improving their fitness and care needs compared to those in the control group. This was evidenced by significantly better results in the control group in both within-group and between-group comparisons after six months post-stroke. Additionally, comparisons between the groups after six months post-stroke showed that patients in the control group had significantly better scores in RS, indicating a better ability to carry out activities of daily living, although no significant within-group changes were observed.

Our results surprisingly revealed that patients who suffered a stroke and participated in exercises, including neurofunctional exercises such as PNF and NDT, had no significant improvements in the Barthel Index and Rankin scale scores six months post-stroke compared to those who only engaged in regular physical exercises at home. These results do not align with previous studies, in which the PNF group showed more improvement than the task-specific group [27]. This suggests that PNF exercises may be not effective in promoting neuroplasticity and enhancing functional activities, opposite to previous findings [27]. Moreover, our results also do not align with studies [28,29] suggesting the benefits of Bobath NDT in enhancing functionality and gait in post-stroke rehabilitation patients compared to traditional rehabilitation methods.

PNF and Bobath NDT are designed to specifically target and enhance the neuroplasticity of the brain [30]. These therapies focus on functional movements and activities that are closely related to daily living tasks, thereby promoting the relearning of motor skills that are essential for independence [31]. The proprioceptive stimuli provided in PNF involve dynamic and reciprocal movements that enhance muscle strength, coordination, and proprioception [32]. This aids in the restoration of normal movement patterns and reduces abnormal muscle tone, which is a common issue in stroke patients [33].

Bobath NDT, on the other hand, emphasizes the importance of postural control and the facilitation of normal movement patterns through guided hands-on techniques [34]. It aims to inhibit abnormal reflexes and tone while encouraging normal movement sequences [35]. By focusing on functional tasks and sensory input, Bobath NDT may help in retraining the brain to control the body more effectively [36]. 

Regular physical exercises, although beneficial for general health and muscle conditioning, do not provide the same level of targeted neural stimulation as neurofunctional exercises [30]. They typically focus on improving cardiovascular fitness and muscle strength, although possibly integrating the stimulus required to promote neuroplastic changes and functional recovery in stroke patients [37].

The improvements observed in the Barthel Index and Rankin scale scores in control group can thus be attributed to the level of physical exercise and environment in the home [30]. The enhancement in muscle strength and coordination may improve the brain’s ability to control movements through targeted neural rehabilitation [30]. This results in improved functional outcomes and greater independence in daily activities for stroke patients, as the home process can enhance family support and participation in exercises.

Regarding the NIHSS scores, a significant decline was observed in the CG, while the SG showed similar results compared to the baseline status. Patients solely engaging in regular physical exercises may not receive the same level of targeted neural stimulation necessary for promoting recovery at the neurofunctional level [38]. While general physical exercises may confer benefits in muscle strength, they may not effectively address the complex neural deficits characteristic of stroke survivors [39]. Without the specificity and intensity of neurofunctional exercises, these patients may experience a decline in functional abilities over time due to inadequate neural reorganization and compensatory mechanisms [40]. Additionally, individual variability in response to rehabilitation interventions must be considered, as factors such as lesion location, severity, and pre-existing comorbidities can influence treatment outcomes [41]. It is plausible that the group undergoing neurofunctional exercises exhibited heterogeneous responses, with some individuals experiencing significant improvements, while others demonstrated more modest gains, resulting in a lack of statistically significant variation at the group level.

Despite the findings, this study has limitations that should be acknowledged. Some limitations include the lack of randomization and blinding, which may have influenced the scoring outcomes. The study’s duration was limited to six months; longer follow-up periods are required to assess the long-term sustainability of the benefits observed with neurofunctional exercises. Furthermore, this study did not account for the potential impact of variations in the intensity and frequency of the rehabilitation sessions among participants, which could influence the outcomes. Monitoring these parameters in future research would help in understanding the optimal rehabilitation protocols for stroke recovery. Moreover, individual differences such as lesion location, severity, and pre-existing comorbidities were not controlled, which could affect the treatment outcomes. Future studies should consider these variables to better understand their influence on the effectiveness of neurofunctional exercises.

Also, we recognize potential limitations related to the assessment tools employed, specifically the BI and the RS. While both scales are widely used in stroke rehabilitation research, they have inherent shortcomings that must be acknowledged. The Barthel Index is susceptible to ceiling effects, which can limit its sensitivity in detecting functional improvements, particularly in patients with milder impairments who may score near the maximum. Additionally, cultural barriers may influence how patients understand and report their functional abilities, potentially affecting the validity of the results. Similarly, the Rankin Scale, despite being a reliable measure of global disability, may also be influenced by subjective interpretations and cultural differences in self-reporting. These factors could impact the generalizability of our findings. Therefore, it is important to consider these limitations when interpreting the results of our study, as they may influence the overall assessment of rehabilitation outcomes in stroke survivors.

Finally, it is essential to acknowledge the limitations of our study regarding the lack of data on concurrent medications, particularly concerning newer antidiabetic drugs. These medications may exhibit antiarrhythmic effects that could be particularly beneficial for patients with atrial fibrillation, a notable risk factor for ischemic stroke [42]. Given the limited efficacy of neurofunctional exercises in patient rehabilitation, there is a compelling need to explore how these newer antidiabetic agents might contribute to both rehabilitation efforts and secondary prevention strategies in this population. Therefore, we advocate for further studies to evaluate the potential benefits of these drugs in patients with atrial fibrillation, which could enhance our understanding of their therapeutic roles and inform clinical practice.

The clinical implications of our findings highlight the effectiveness of home-based exercise programs in promoting positive outcomes for stroke rehabilitation. Regular participation in these exercises not only improves patients’ abilities to perform daily activities—reflected in enhanced Barthel Index and Rankin Scale scores—but also helps mitigate the decline in cognitive and motor function commonly observed in stroke survivors engaged solely in traditional physical activities. By adopting a well-structured home workout regimen, stroke patients can experience significant improvements in their quality of care, leading to better long-term outcomes and reduced care needs.

Clinicians should actively consider integrating these exercise plans into rehabilitation protocols to optimize recovery and improve the overall well-being of stroke survivors. Additionally, combining home-based exercises with specialized approaches such as PNF may further enhance rehabilitation efforts. PNF exercises focus on dynamic and reciprocal movements that strengthen muscles, improve coordination, and enhance proprioception, effectively restoring normal movement patterns and addressing common issues like abnormal muscle tone in stroke patients. Similarly, NDT emphasizes postural control and normal movement patterns, utilizing guided techniques to inhibit abnormal reflexes and promote functional sequences.

While regular exercises can be beneficial for general health and muscle conditioning, they often lack the targeted neural stimulation essential for addressing the complex deficits resulting from strokes. Therefore, a structured home exercise regimen, supplemented with neurofunctional exercises, can significantly improve stroke patients’ ability to engage in daily tasks, further evidenced by better performance on the Barthel Index and Rankin Scale. This comprehensive approach underscores the need for tailored rehabilitation protocols that consider individual variability in responses to treatment, ultimately fostering recovery and enhancing the quality of life for stroke survivors.

## 5. Conclusions

Based on the findings of the current prospective cohort study, it is evident that stroke patients undergoing neurofunctional exercises did not exhibit substantial improvements in their fitness and care needs compared to those in the control group. This study revealed significantly better outcomes within the control group both individually and in comparison to the neurofunctional exercise group six months post-stroke. Furthermore, assessments between the groups at this stage indicated superior scores in the control group for RS, reflecting enhanced proficiency in daily activities. These results underscore the need for further research to explore more effective rehabilitation strategies tailored to meet the complex needs of stroke survivors.

## Figures and Tables

**Table 1:** Descriptive statistics (mean ± standard deviation) of the demographic information and the rehabilitation scales between groups.

	SG (*n* = 51)	CG (*n* = 51)	Between Group Comparisons	Effect Size (r)
Men (*n*)	20	26		
Women (*n*)	31	25		
Age (years)	72.2 ± 9.9	69.5 ± 10.8		
Barthel index (A.U.)				
Baseline	43.0 ± 35.5	67.9 ± 34.0	*p* = 0.001	0.320
Median	30.0	80.0		
Interquartile range	70.0	55.0		
Post 6 months	63.5 ± 32.9	78.1 ± 28.0	*p* = 0.021	0.229
Median	70.0	90.0		
Interquartile range	55.0	25.0		
Post-pre difference (%)	47.7	15.0		
Ranking scale (A.U.)				
Baseline	3.4 ± 1.3	2.5 ± 1.3	*p* < 0.001	0.331
Median	4.0	2.0		
Interquartile range	2.0	2.0		
Post 6 months	3.3 ± 1.3	2.6 ± 1.4	*p* = 0.019	0.232
Median	3.0	3.0		
Interquartile range	2.0	2.0		
Post–pre difference (%)	−2.9	4.0		
NIHSS (A.U.)				
Baseline	6.5 ± 5.3	3.1 ± 3.9	*p* = 0.001	0.436
Median	5.0	2.0		
Interquartile range	6.0	2.0		
Post 6 months	5.9 ± 6.0	5.8 ± 6.3	*p* = 0.755	0.031
Median	4.0	4.0		
Interquartile range	6.0	5.0		
Post–pre difference (%)	−9.2	87.1		

NIHSS: National Institutes of Health Stroke Scale; A.U.: arbitrary units. SG: experimental group; CG: control group.

## Data Availability

The data can be provided upon a reasonable request to the corresponding author.
